# Identification of miRNAs Enriched in Extracellular Vesicles Derived from Serum Samples of Breast Cancer Patients

**DOI:** 10.3390/biom10010150

**Published:** 2020-01-16

**Authors:** Patricia M. M. Ozawa, Evelyn Vieira, Débora S. Lemos, Ingrid L. Melo Souza, Silvio M. Zanata, Vânia C. Pankievicz, Thalita R. Tuleski, Emanuel M. Souza, Pryscilla F. Wowk, Cícero de Andrade Urban, Flavia Kuroda, Rubens S. Lima, Rodrigo C. Almeida, Daniela F. Gradia, Iglenir J. Cavalli, Luciane R. Cavalli, Danielle Malheiros, Enilze M. S. F. Ribeiro

**Affiliations:** 1 Department of Genetics, Federal University of Paraná, Curitiba 81531-980, Brazil; paty.mih@gmail.com (P.M.M.O.); evelynvieira1995@gmail.com (E.V.); deboraslemos@gmail.com (D.S.L.); rodrigocout@gmail.com (R.C.A.); danielagradia@gmail.com (D.F.G.); cavalli@ufpr.br (I.J.C.); 2 Department of Cell and Molecular Biology, Federal University of Paraná, Curitiba 81531-980, Brazil; ingridlms@yahoo.de (I.L.M.S.); smzanata@ufpr.br (S.M.Z.); 3 Department of Biochemistry and Molecular Biology, Federal University of Paraná, Curitiba 81531-980, Brazil; vaniacarla@gmail.com (V.C.P.); thalituleski@hotmail.com (T.R.T.); souzaem@ufpr.br (E.M.S.); 4 Carlos Chagas Institute - Fiocruz-Paraná, Curitiba 81350-010, Brazil; pryscilla@wowk.com.br; 5 Positivo University Medical School, Curitiba 81280-330, Brazil; 6 Nossa Senhora das Graças Hospital Breast Unit, Curitiba 80810-040, Brazil; flaviakuroda@hotmail.com (F.K.); rsilveiralima@uol.com.br (R.S.L.); 7 Department of Biomedical Data Sciences, Molecular Epidemiology, Leiden University Medical Center, 2300 R Leiden, The Netherlands; 8 Department of Oncology, Lombardi Comprehensive Cancer Center, Georgetown University, Washington, DC 20057, USA; lrc@georgetown.edu; 9 Research Institute Pelé Pequeno Príncipe, Faculdades Pequeno Príncipe, Curitiba 80230-020, Brazil

**Keywords:** extracellular vesicles, circulating microRNAs, RNA-seq, miRNA, liquid biopsy

## Abstract

MicroRNAs derived from extracellular vesicles (EV-miRNAs) are circulating miRNAs considered as potential new diagnostic markers for cancer that can be easily detected in liquid biopsies. In this study, we performed RNA sequencing analysis as a screening strategy to identify EV-miRNAs derived from serum of clinically well-annotated breast cancer (BC) patients from the south of Brazil. EVs from three groups of samples (healthy controls (CT), luminal A (LA), and triple-negative (TNBC)) were isolated from serum using a precipitation method and analyzed by RNA-seq (screening phase). Subsequently, four EV-miRNAs (miR-142-5p, miR-150-5p, miR-320a, and miR-4433b-5p) were selected to be quantified by quantitative real-time PCR (RT-qPCR) in individual samples (test phase). A panel composed of miR-142-5p, miR-320a, and miR-4433b-5p distinguished BC patients from CT with an area under the curve (AUC) of 0.8387 (93.33% sensitivity, 68.75% specificity). The combination of miR-142-5p and miR-320a distinguished LA patients from CT with an AUC of 0.9410 (100% sensitivity, 93.80% specificity). Interestingly, decreased expression of miR-142-5p and miR-150-5p were significantly associated with more advanced tumor grades (grade III), while the decreased expression of miR-142-5p and miR-320a was associated with a larger tumor size. These results provide insights into the potential application of EVs-miRNAs from serum as novel specific markers for early diagnosis of BC.

## 1. Introduction

Extracellular vesicles (EVs) are known to be actively secreted in body fluids by many types of cells [1] with the purpose of signaling communication [2]. Their content includes microRNAs (miRNAs) [3], which are small noncoding RNAs with 21 to 25 nucleotides involved in gene regulation [4] and are known to reflect the physiologic and pathologic state of the organism. Several studies have shown the potential of miRNAs as biomarkers of disease [5,6], including breast cancer (BC) [7].

The most recent Globocan report reported BC as the most frequently diagnosed cancer in females and as the leading cause of death by cancer in women worldwide, reaching around 626,679 deaths in 2018 [8]. In Brazil, BC is considered the second most lethal cancer among women [9]. The classification of BC adopted in clinical evaluation is based on immunohistochemistry expression and stratifies BC in luminal A (ER^+^ (estrogen receptor positive), PR^+^ (progesterone receptor positive), HER2^−^ (human epidermal growth factor receptor 2 negative), Ki-67 ≤ 14), luminal B (ER^+^, PR^+/−^, HER2^+/−^, Ki-67 > 14), HER2 overexpression (ER^−^, PR^−^, HER2^+^), and triple-negative (ER^−^, PR^−^, HER2^−^) [10]. These BC subtypes have a distinct association with patients’ prognosis and outcomes, which impact treatment choices. The luminal A (LA) subtype confers the best prognosis to patients, while triple-negative BC (TNBC) is considered the poorest prognosis among all BC subtypes [11].

Early detection is the best strategy to improve patient outcomes. Several studies have suggested that miRNAs could be suitable biomarkers [12,13,14,15] for early diagnosis of cancer, notably for its remarkable stability in liquid biopsies. In this context, although in its early stages, the use of EV-derived miRNAs (EV-miRNAs) from serum and plasma as a biomarker for BC have shown promising results [7,14,16,17,18,19]. Among the EV-miRNAs described to be overexpressed in BC compared to control are miR-21, miR-1246 [7], and the miR-106a–363 cluster [14]. Interestingly, a recent report showed that detection of miR-103, miR-191, and miR-195 in EVs associated with red blood cells can also be used to distinguish BC patients from controls in plasma [20]. Additionally, expression of EV-miRNAs has been described with other parameters, such as miR-373 being overexpressed in TNBC compared to luminal carcinomas [16], and miR-340-5p with BC recurrence [18].

The search for a non-invasive and novel biomarker for BC diagnosis led us to investigate the EV-miRNAs identified by RNA sequencing (RNA-seq) analysis. In this study, we used EV-miRNAs isolated from the serum of clinically well-annotated BC patients, luminal A (LA) and triple-negative (TNBC), and healthy controls (CT) from the south of Brazil. RNA-seq data and subsequent analysis by quantitative real-time PCR (RT-qPCR) performed in four selected EV-miRNAs (miR-142-5p, miR-150-5p, miR-320a, and miR-4433b-5p) revealed that the combination of miR-142-5p, miR-320a, and miR-4433b-5p distinguished BC patients from controls with high power, while the combination of miR-142-5p and miR-320a distinguished LA patients from CT. Importantly, lower expression of miR-142-5p and miR-150-5p was associated with higher tumor grade (III) and decreased expression of miR-142-5p and miR-320a with larger tumor sizes (< 20 mm). Overall, these EV-miRNAs presented high accuracy in identifying BC patients, showing the potential application of EV-miRNAs as liquid biomarkers for BC diagnosis.

## 2. Materials and Methods

### 2.1. Breast Cancer Patients and Controls

This study was approved by the Ethical Committee in Research from the Health Sciences Unit of the Federal University of Paraná (UFPR), CAAE: 67029617.4.0000.0102, approval: 2033689, acceptance date: 26 April 2017. The experiments were carried out in accordance with the principles of the Declaration of Helsinki of 1975. All individuals signed a written informed consent agreement for the use of their samples for research purposes. Serum samples from 31 patients with invasive ductal carcinoma (16 LA and 15 TNBC) were collected before surgery and any therapy at Nossa Senhora das Graças Hospital, Curitiba, Brazil. Patients’ pathological reports were accessed without identifiers to categorize the samples according to the immunohistochemical classification. Patients’ clinicopathological characteristics are listed in Table 1. Serum samples from 16 healthy subjects (control (CT)) with no personal record of BC were collected from volunteers at UFPR. The mean age of this group was 57.4 ± 7.1 (range from 47 to 77 years old). All samples were collected using an 8.5 mL vacutainer serum tube with gel (BD, Franklin Lakes, NJ, USA) and centrifuged at 770× *g* for 10 min. The supernatant was collected and stored at −80 °C. 

### 2.2. Study Design

**Figure 1:**
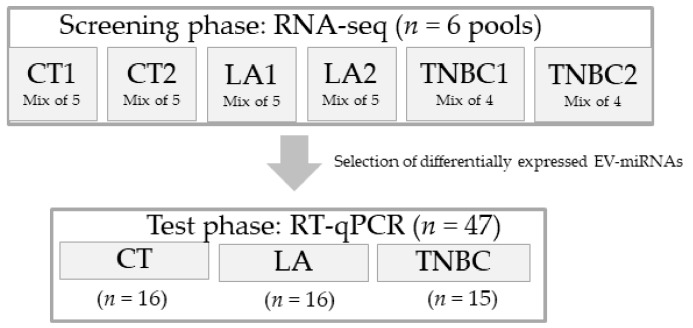
Schematic diagram of the workflow of this study. This study was designed in two phases. During the screening phase we performed RNA sequencing (RNA-seq) analysis in six pools of sample composed of two sample per group (Control (CT), Luminal A (LA), and Triple-negative (TNBC). During the test phase, a selection of EV-miRNAs found to be differentially expressed in the RNA-seq analysis was chosen to be tested in 47 individual patient samples’ by quantitative real-time PCR (RT-qPCR).

This study was designed in two phases (Figure 1): screening and test phases. For the screening phase, six pooled samples of EV-miRNAs, composed of two pools of each group (CT1 (*n* = 5), CT2 (*n* = 5), LA1 (*n* = 5), LA2 (*n* = 5), TNBC1 (*n* = 4), and TNBC2 (*n* = 4)) were sequenced via RNA-seq. Each group was composed of a mix of EV-miRNAs from individuals with matching ages. For all samples, EV isolation and EV-miRNA extraction were performed separately and mixed only after the EV-miRNA’s extraction. For the test phase, EV-miRNAs from individual samples (CT (*n* = 16), LA (*n* = 16), and TNBC (*n* = 15)) were submitted for analysis of four selected EV-miRNAs: miR-142-5p, miR-150-5p, miR-320a, and miR-4433b-5p. These miRNAs were found to be differentially expressed (DE) among the groups analyzed by RNA-seq (CT versus CA, CT versus LA, CT versus TNBC, and LA versus TNBC). They also had significant *p*-values and involvement in cancer-related pathways.

### 2.3. EV Isolation

For the EV isolation, we used different amounts of serum from patients and control: 1 mL for the screening phase and 300 µL for the test phase and EV characterization. The isolations were performed using the Total Exosome Isolation Reagent from the Serum (Invitrogen, Carlsbad, CA, USA) precipitation kit, according to the manufacturer’s instructions. EVs isolated for RNA extraction, nanoparticle tracking analysis (NTA), and transmission electron microscopy (TEM) analysis were resuspended in 200 µL of 1× filtered (0.22 µm) phosphate-buffered saline (PBS) and immediately proceeded to downstream processing. EVs isolated for Western blotting (WB) analysis were eluted in 150 µL of lysis buffer and stored at −20 °C.

### 2.4. EV Characterization

EV characterization was performed following the requirements of the International Society of Extracellular Vesicles [21]. NTA was performed using the Nano-Sight LM10 (Malvern Panalytical, Malvern, UK) instrument at Carlos Chagas Institute Fiocruz-PR, Curitiba, Brazil, to quantify and characterize EVs’ sizes. In this analysis, five videos of 30 s were recorded using five samples from each group (CT, LA, and TNBC). The average size from these videos was used to assess the size distribution of EVs. 

TEM was performed to analyze EVs’ sizes and shapes. Briefly, approximately 7 µL of EVs (eluted in PBS) were fixed on 4% paraformaldehyde and added on a Formvar carbon-coated copper grid, followed by 2% uranyl treatment for 1 min. The EVs were then evaluated under a JEOL 1200EX II transmission electron microscope (JEOL, Akishima, Tokyo, JP) at 110 V, available at the Electron Microscopy Center, Federal University of Paraná (UFPR) (Curitiba, Brazil).

For WB analysis, EVs were quantified using Bradford assay (Bio-Rad, Hercules CA, USA) and 30 µg of each sample (control-EV, control supernatant, cancer-EV, and cancer supernatant) was loaded and run under non-reducing conditions. We used primary antibodies specific for CD9 (cat# 10626D) and CD63 (cat# 10628D) (Invitrogen, CA, USA) (1:1000) and Goat anti-Mouse horseradish peroxidase (HRP) conjugated secondary antibody (cat# A16066) (Invitrogen, CA, USA) (1:2,000). The proteins were detected using SuperSignal™ West Pico PLUS Chemiluminescent Substrate (Thermo Fisher Scientific, Waltham, MA, USA) and captured with Amersham Hyperfilm ECL (GE Healthcare Life Science, Marlborough, MA, USA). Considering that the antibodies utilized are commonly used as exosomal markers but can also be present in other types of EVs, we adopted the general term EVs in this study.

### 2.5. RNA Extraction

EV-miRNAs were extracted using miRVana™ miRNA Isolation Kit (Ambion, Waltham, MA, USA) and modified with the addition of Trizol (Invitrogen, CA, USA) in substitution to phenol. Also, 25 pmol spike-in cel-miR-39 (Qiagen, Germantown, MD, USA) was added as an exogenous control. Samples were eluted at a final volume of 20 µL and the concentration and purity was determined using a Nanodrop 2000 spectrophotometer (Thermo Fisher Scientific, CA, USA) and Bioanalyzer (Agilent Inc., Santa Clara, CA, USA). Samples were stored at −80 °C until further analysis. All samples were extracted separately and later mixed into pools for the screening phase analysis.

### 2.6. Profiling of Small RNA Cargo of EVs

Characterization and quantification of the EV-miRNA profile from serum samples of patients and controls were performed by RNA-seq. The Libraries were constructed using the Ion total RNA-seq kit v2 for Whole Transcriptome Library (Life Technologies, Carlsbad, CA, USA) following the manufacturer’s instructions for small RNA libraries. Six pools (CT1, CT2, LA1, LA2, TNBC1, and TNBC2) were sequenced on the Next Generation sequencing platform Ion Proton™ System^®^ (Thermo Fisher Scientific, MA, USA) platform using the Ion PI Template OT2 200 Kit v3 and the Ion PI Sequencing 200 Kit v3 (Life Technologies) following the manufacturer’s instructions. Data alignment and mapping were performed with the MiRMaster platform (www.ccb.uni-saarland.de/mirmaster) [22]. Kyoto Encyclopedia of Genes and Genomes (KEGG) pathway analyses were performed using Diana Tools mirPath v.3 online, selected for the Tarbase database [23]. Cancer group (CA) was defined by the combined analysis of the LA and TNBC groups of patients. Raw and processed data were uploaded to the Gene Expression Omnibus (GEO) database, accession number: GSE141326.

### 2.7. RT-qPCR

A subset of EV-miRNAs were selected for further analysis in a larger cohort of BC patients (*n* = 31) and controls (*n* = 16). These samples included individuals used in the screening phase. The selection of EV-miRNAs for RT-qPCR analysis was based on the following parameters: highest statistical significance in multiple comparisons as observed in the RNA seq analysis and involvement in pathways related to cancer according to KEGG pathway analysis (Diana tools, mirPath v.3).

The complementary DNA (cDNA) synthesis was performed using TaqMan MicroRNA Reverse Transcription Kit (Applied Biosystems, city, CA, USA), as follows: a mixture of 1.25 mM deoxyribonucleotide triphosphate (dNTPs) (with Deoxythymidine triphosphate (dTTP)), 3.75 U/µL of MultiScribe™ Reverse Transcriptase, 1x of Reverse Transcription Buffer, 0.25 U/µL of RNAse inhibitor, 0.125× of each primer, 10 µL of total RNA extracted, for a final volume of 20 µL. Primers used were: (has-miR-142-5p (ID: 002248), has-miR-150-5p (ID: 000473), has-miR-320a (ID: 002277), and has-miR-4433b-5p (ID: 466345_mat) with cel-miR-39 (ID: 000200)). The RT-PCR reaction was performed at 25 °C for 10 min, 37 °C for 2 h, and 85 °C for 5 min, on the Eppendorf 5331 MasterCycler Gradient Thermal Cycler (Eppendorf, Hamburg, Germany).

Next, for RT-qPCR, cDNA samples were diluted 1:5, 9 µL of this dilution was added to 1× TaqMan Universal PCR Master Mix II (no uracil-N-glycoslyase (UNG)), 1× TaqMan Small RNA assay (individually), for a final volume of 20 µL, and distributed in triplicates of 5 µL each, in a 384 well plate. A cDNA negative control was included. qPCR assays were performed using ViiA 7 Real-Time PCR System (Applied Biosystems, CA, USA) with the following protocol: 50 °C for 5 min, 95 °C for 10 min, 40 cycles of 95 °C for 15 s, and 60 °C for 60 s. The threshold standard deviation (SD) adopted for the intra-assay and inter-assay replicates was 0.5. The relative quantity (RQ) of miRNA expression was calculated using the comparative cycle threshold (2^−ΔΔCt^) method [24] normalized to cel-miR-39 levels (exogenous control used to standardize miRNA expression). BC cell line BT-474 had detectable expression levels of all miRNAs and was chosen to be used as a positive control for all qPCR plates. All calculations were performed using QuantStudio Real-Time PCR Software v1.3 (Applied Biosystems, CA, USA).

### 2.8. Statistical Analysis

Differentially expressed (DE) miRNAs observed in the RNA-seq analysis were identified using the package DESeq2 [25] in R studio [26,27]. This package performs an internal normalization based on the median of the ratios of observed counts [28]. The Benjamini and Hochberg method was used for multiple comparisons, with an adjusted *p*-value of <0.05 being considered significant. For RT-qPCR analysis, the data was calculated based on Relative Quantification (RQ) = 2^−ΔΔCt^ values in triplicate samples. All the data (including clinicopathological data) were initially analyzed via D’Agostino-Pearson omnibus and the Shapiro-Wilk normality tests, as well as the Brown–Forsythe test to check for variance homogeneity. When the data did not fit either of these tests, non-parametric tests were performed. The comparison of miRNA expression between two groups was calculated using the Mann–Whitney test. Groups with three parameters were analyzed using the Kruskal–Wallis test, followed by the Dunn’s multiple comparisons test. The adjusted *p*-value of <0.05 was considered significant. Receiver operating characteristic (ROC) curves were calculated based on RQ values. For combined ROC curves, a binary logistic regression was calculated using IBM SPSS Statistics (IBM SPSS Statistics Inc, Armonk, NY, USA). The true positive rate (sensitivity) versus the false positive rate (1-specificity) were plotted at various threshold settings, with the optimal cutoff threshold calculated using Youden’s index (highest sensibility plus specificity). Efficiency values were determined for the miRNAs (individually and combined), as previously described [29].

All statistical analyses were performed using GraphPad Prism 6 (GraphPad Software Inc., San Diego, CA, USA), except for the binary logistic regression analysis, which was calculated using IBM SPSS Statistics.

## 3. Results

### 3.1. EVs’ Isolation and Characterization

**Figure 2:**
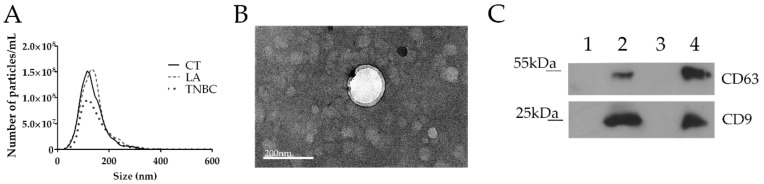
Extracellular vescile (EV) characterization. (**A**) Nanoparticle tracking analysis (NTA) showing a peak between 100–200 nm for the control (CT), luminal A (LA), and triple negative (TNBC) groups. (**B**) Transmission electron microscopy (TEM) image of EVs from cancer patient showing a size corresponding to NTA results. Size bar = 200 nm. (**C**) Western blotting (WB) analysis showing strong protein expression of CD9 and CD63 on EVs from control (2) and cancer (4), when compared with corresponding serum supernatant EV-depleted from control (1) and cancer (3).

Small EVs with exosome characteristics were isolated from five samples from each group: CT, LA, and TNBC, showing a mean size of approximately 140 nm (Figure 2A). No statistically significant differences were observed in the mean, mode, and concentration of the EVs among the groups of patients and controls in the NTA analysis (data is not shown). TEM analysis showed that EVs’ size corresponded to the NTA results (Figure 2B). WB analysis showed enrichment of exosome-related proteins, CD9 and CD63, in the EVs isolated from both cancer and control samples when compared with EV-depleted supernatant (Figure 2C). These results indicate that our EV population is enriched in particles that might correspond to exosomes.

### 3.2. Differentially Expressed EV-miRNAs Identified by RNA-seq are Suitable Biomarkers for Diagnosis of BC and Its Subtypes

The six pooled samples (cancer and control groups) were sequenced using the IonTorrent platform. The number of total raw reads per group (CT, LA, and TNBC) were: 24,008,940, 20,256,190, and 21,511,347, respectively. The reads were mapped to the human genome database (hg38) and the percentage of homologous reads was: 12%, 34%, and 49%, respectively. Comparison of the reads to the miRbase v21 database showed a mapping percentage of 3%, 5%, and 6%, respectively.

The screening phase showed several EV-miRNAs where DE between the groups can be potential biomarkers for disease (CT versus CA (*n* = 22), CT versus LA (*n* = 19), and CT versus TNBC (*n* = 30)), and for subtype differentiation (LA versus TNBC (*n* = 7)) (Supplementary Table S1). The top seven EV-miRNAs DE among the groups analyzed are shown in Table 2. Interestingly, KEGG pathway analyses showed that all EV-miRNAs found DE in the four analyses are involved in pathways related to cancer, such as the “proteoglycan in cancer”, “microRNAs in cancer”, and “pathways in cancer” pathways (Supplementary Table S2).

Four miRNAs (miR-142-5p, miR-150-5p, miR-320a, and miR-4433b-5p) were selected to be quantified in the test phase by RT-qPCR to evaluate the reproducibility of expression levels in a larger cohort of samples (individual samples). The selection of these miRNAs was based on their *p*-values and on the KEGG pathway analysis (Table 3) that showed their strong association with cancer signaling pathways previously mentioned. The exception was miR-4433b-5p, which was selected solely based on its high *p*-value, even though it has no experimentally validated record in association with tumorigenic processes.

### 3.3. EV-miRNAs Expression in Individual Serum Samples

**Figure 3:**
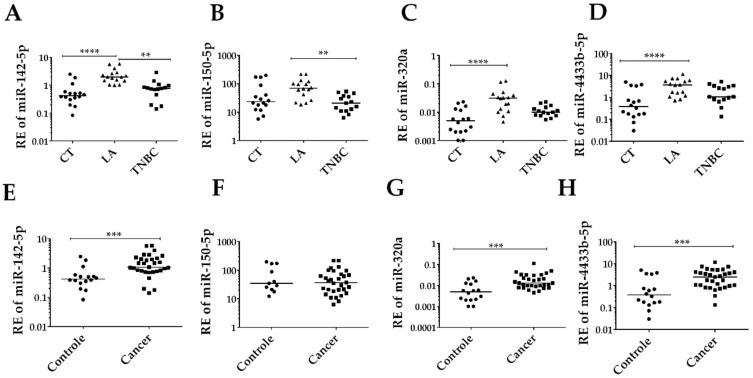
Expression of the selected group of EV-miRNAs according to BC diagnosis and subtypes. (**A**) Significantly increased relative expression (RE) of miR-142-5p in Luminal A (LA) patients compared to Triple-negative (TNBC) patients (*p* < 0.0001), and control (CT) (*p* < 0.01). (**B**) miR-150-5p RE level is significantly higher in LA compared to TNBC patients (*p* < 0.01). (**C**) miR-320a and (**D**) miR-4433b-5p levels are higher in LA patients compared to CT (*p* < 0.0001 for both comparisons). (**E**) Significant overexpression of miR-142-5p in cancer (LA + TNBC) patients compared to CT (*p* < 0.001). (**F**) No difference in miR-150-5p level between cancer and control. (**G**) Significant overexpression of miR-320a and (**H**) miR-4433b-5p in cancer patients in comparison to controls (*p* < 0.001 for both). Cel-miR-39 was used as the exogenous control. BT-474 was used as a calibrator in all plates. The y-axis values are in log10, for a better presentation of the data. Analyses were calculated based on relative quantification (RQ) = 2^−ΔΔCt^ values. ** *p* < 0.01; *** *p* < 0.001; **** *p* < 0.0001.

Of the four EV-miRNAs selected above and analyzed in the individual serum samples (31 BC patients and 16 CT), three (miR-142-5p, miR-320a, and miR-4433b-5p) presented a significant difference in the expression level between CT and BC patients (Figure 3A,D). These three EV-miRNAs were overexpressed in cancer patients when compared to the CT group. Within the BC subtypes, the LA subtype showed a statistically significant increase compared to the TNBC subtype (Figure 3E–H).

### 3.4. Association of EV-miRNAs’ Expression with the Clinicopathological Parameters of BC Patients

**Figure 4:**
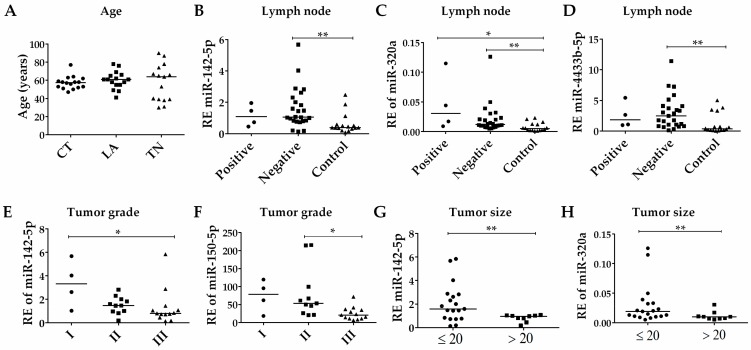
Comparison of EV-miRNAs expression to clinicopathological data. (**A**) No difference was observed between the patients’ ages for the evaluated groups: control (CT), Luminal A (LA), and Triple-negative (TNBC). (**B**) Significant down-relative expression (RE) of miR-142-5p in controls compared to patients negative for lymph node metastasis (*p* < 0.01). (**C**) Significant increase in levels of miR-320a in patients with BC, independent of lymph node metastasis status (*p* < 0.05 for both). (**D**) Patients without lymph node metastasis still present higher levels of miR-4433b-5p compared to controls (*p* < 0.01). (**E**) Significant reduction in miR-142-5p (*p* < 0.05) between tumor grade I and III. (**F**) No significant alteration of miR-150-5p among tumor grades. (**G**) Bigger tumors (>20 mm) have decreased levels of miR-142-5p (*p* < 0.01), and (**H**) miR-320a (*p* < 0.01). * *p* < 0.05, ** *p* < 0.01. Analysis were calculated based on relative quantification (RQ) values.

The association of patients clinicopathological parameters (Table 1) with the expression data was measured for the four selected EV-miRNAs. No significant differences in age were observed between the CT, LA, and TNBC groups (Figure 4A). Additionally, there was no difference in EV-miRNA expression levels between age groups (<50 and ≥50) (data not shown). There were no significant differences in EV-miRNAs’ expression in BC patients according to the status of lymph node metastasis. Interestingly, the expression levels of the EV-miRNAs miR-142-5p, miR-320a, and miR-4433b-5p in patients with no lymph node metastasis differed from the controls (Figure 4B,D). A significant decrease in the expression levels of miR-142-5p (Figure 4E) and miR-150-5p (Figure 4F) was observed with more advanced tumor grades (grade III). Additionally, low expression levels of miR-142-5p (Figure 4G) and miR-320a (Figure 4H) were observed in larger tumors (>20 mm) when compared to tumors of smaller sizes (≤20 mm). These results suggest that even patients with no lymph node metastasis have higher levels of miR-142-5p, miR-320a, and miR-4433b-5p compared to control. Additionally, the expression levels of miR-142-5p and miR-150-5p are lower in advanced tumor grades, while smaller tumors are associated with higher levels of miR-142-5p and miR-320a.

### 3.5. Diagnostic Potential of EV-miRNAs in Serum Samples 

**Figure 5:**
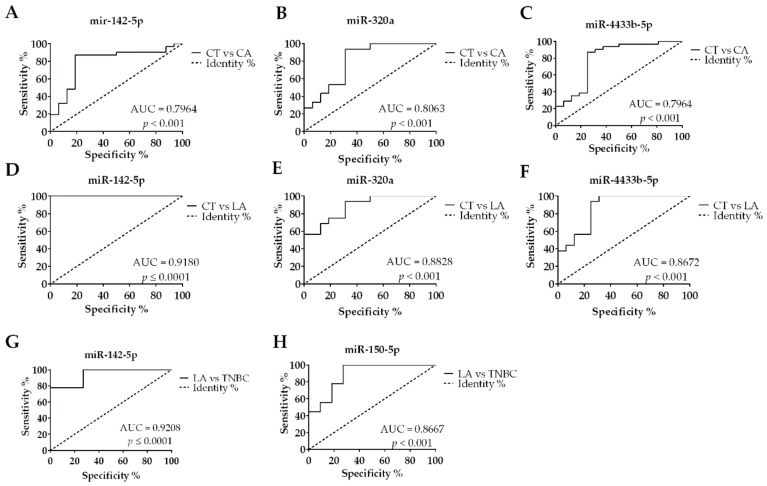
Individual receiver operating characteristic (ROC) curves from EV-miRNAs tested by RT-qPCR. (**A**) BC diagnosis accuracy calculated for miR-142-5p, (**B**) miR-320a, and (**C**) miR-4433b-5p. (**D**) Accuracy to diagnose Luminal A (LA) patients over Controls (CT) using miR-142-5p, (**E**) miR-320a, and (**F**) miR-4433b-5p. (**G**) Capacity to discriminate LA over Triple-negative (TNBC) patients using miR-142-5p and (**H**) miR-150-5p. ROC curves calculated based on relative quantification (RQ) values. Only area under the curve (AUC) > 0.79 are shown.

**Figure 6:**
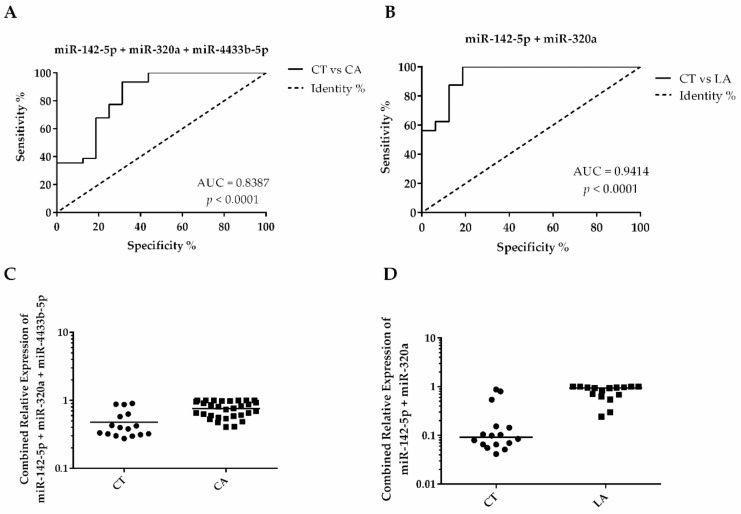
Combined ROC curves and scatter plots indicating diagnosis potential of EV-miRNAs panels. (**A**) ROC curve calculated combining miR-142-5p, miR-320a, and miR-4433b-5p to diagnose BC. (**B**) Panel combining miR-142-5p and miR-320a showing excellent accuracy to diagnose LA patients. (**C**) Control (CT) and cancer (CA) distribution after binary logistic regression analysis combining relative quantification (RQ) values of miR-142-5p, miR-320a, and miR-4433b-5p. (**D**) CT and luminal A (LA) distribution after binary logistic regression combining RQ values of miR-142-5p and miR-320a.

The EV-miRNAs’ diagnostic potential for BC and its subtypes was tested by the construction of individual ROC curves (Figure 5A,C). We analyzed only the statistically significant comparisons, based on our findings in Figure 3. From the four EV-miRNAs analyzed, miR-320a presented the best individual discriminatory power in the comparison of BC versus CT (Figure 5B), presenting an area under the curve (AUC) of 0.8063, with a sensitivity of 93.33% and specificity of 68.75%, for the optimal cutoff value of 0.0060 (Table 4). Interestingly, the discriminatory accuracy is improved when combined with the expression levels of miR-142-5p and miR-4433b-5p, which also presented good AUC values (Figure 5A and C, respectively). The combined AUC of these miRNAs (miR-320a, miR-142-5p, and miR-4433b-5p) improved to 0.8387 (Figure 6A), while the sensitivity and specificity values remained almost the same (93.55% and 68.75%, respectively, for an optimal cutoff value of 0.4504). Taken together, these results indicate that a panel composed by these three miRNAs could be used to diagnose BC patients from serum samples with high accuracy.

The analysis of the ROC curves between BC subtypes showed that miR-320a (AUC = 0.8828, cutoff value of 0.0065, 93.75% sensitivity, and 68.75% specificity), and miR-4433b-5p (AUC = 0.8672, cutoff value of 0.7743, 93.75% sensitivity, and 75% specificity) can be used as indicators in discriminating LA and TNBC subtypes in patients. On the other hand, miR-142-5p presented the best AUC value to identify LA patients when compared to CT (AUC = 0.9180, cutoff value of 0.7926, presenting 100% sensitivity, and 81.25% specificity) and TNBC patients (AUC = 0.9208, cutoff value of 0.6435, presenting 87.10% sensitivity, and 81.25% specificity). Interestingly, a panel combining miR-142-5p and miR-320a (Figure 6B) improved the AUC to 0.9410, showing 100% sensitivity and 93.80% specificity, for an optimal cutoff value of 0.087). The miR-150-5p was the only miRNA not capable of detecting BC with accuracy; however, it showed a suitable AUC value to discriminate between LA and TNBC subtypes (AUC = 0.8667, sensitivity of 80% and specificity of 75%, for a cutoff = 39.3800). Altogether, these results show that the EV-miRNAs selected, individually or in combination, can distinguish BC patients from CT, in addition to being useful in distinguishing between the LA and TNBC subtypes from one another.

## 4. Discussion

Biomarkers for breast cancer diagnosis in liquid biopsies have been extensively studied over the years [13,14,15,30,31]. Several of these studies focused on circulating miRNAs (c-miRNAs) due to their high stability and easy accessibility in biofluids specimens [32]. Several miRNAs have been identified as potential biomarkers for BC, individually [33,34,35,36,37] or as panels [7,13,14,15,38,39,40,41]. However, clinical validation of these c-miRNAs has been a challenge considering the divergence found among studies in relation to the optimal selection of c-miRNAs and their specificity for BC [42].

Nonetheless, new studies have been focusing on the analysis of EV-miRNAs cargoes as potential biomarkers for BC diagnosis [7]. The loading of miRNAs in EVs has been shown to be a controlled process, with the selection of the miRNAs cargoes being defined according to the cell of origin, also being representative of the parental tumor cells in cancer [42,43]. Therefore, EV-miRNAs have emerged as potential diagnostic and prognostic markers that can be easily detected using non-invasive procedures, such as liquid biopsies. In this study, we performed RNA-seq analysis as a screening strategy to identify miRNAs enriched in EVs derived from serum samples of BC patients. To the best of our knowledge, this is the first study to perform next-generation sequencing (NGS) to determine the miRNA expression profile of EVs derived from BC patients. Most studies in the literature target specific miRNAs by RT-qPCR, or perform microarray analysis, which are restricted to the detection of miRNAs selected from genomic databases. The advantage of using NGS resides in the measurement of relative expression levels of miRNAs, generating the unique possibility of identifying novel and not commonly studied miRNAs, overcoming the coverage limitations of the array-based approaches [44]. However, NGS demands high amounts of RNA as input and the extraction of EV-miRNAs renders low amounts of RNA. Therefore, in this study, we adopted the utilization of pooled samples to represent groups of BC patients, to obtain enough RNA to yield reproducible reads.

The miRNA analysis in the screening phase of this study, performed in six pooled serum samples (cancer and control groups), showed a significant number of DE EV-miRNAs in all group comparisons (varying from seven to 30 EV-miRNAs, according to the group comparison). The KEGG pathway analysis of all DE EV-miRNAs in the groups confirmed the involvement of these EV-miRNAs in pathways related to cancer. This data suggests that these EV-miRNAs are representative of tumor cells. The expression of four of the observed DE miRNAs (miR-142-5p, miR-150-5p, miR-320a, and miR-4433b-5p) selected were further validated by RT-qPCR (test phase) in a larger number of samples. These miRNAs have been previously characterized by their oncogenic and/or tumor suppressor function in other types of cancers, including BC [29,45,46], except for miR-4433-5p, that is a new miRNA with no description in cancer so far.

In the test phase, miR-320a was shown to be overexpressed in the CA and LA groups compared to the CT group. However, the expression of miR-142-5p was significantly higher in the LA group compared to the CT and TNBC groups. Similarly, miR-4433b-5p was overexpressed in the LA group compared to the CT group, but not between LA versus TNBC groups, and miR-150-5p was only DE between the LA and TNBC groups. Interestingly, the analysis of the clinical data from these patients showed that the decreased expression of miR-142-5p and miR-150-5p were significantly associated with more advanced tumor grades, while the decreased expression of miR-142-5p and miR-320a were associated with larger tumor size.

Expression of miR-142-5p has already been associated with TNBC [46], with its overexpression also being associated with lymph node metastasis [47,48], although this was not seen in the cases of this study. Importantly, a recent study described this miRNA as having an oncogenic role in BC [48], which is in line with the findings of this study. Significantly high levels of miR-142-5p were observed in BC tissue compared to the adjacent tissue, and functional studies in MDA-MB-231 cells showed that inhibition of miR-142-5p caused decrease in its proliferation and induced its apoptosis, probably by increase of *PTEN* expression [48].

The higher expression level of miR-150-5p has been described in BC subtypes, mostly in TNBC [29,49,50], and also in basal I [51] subtypes. It has been described as a good prognostic biomarker for patients with HER2-positive BC [52,53]. Overexpression of miR-150-5p has been described in TNBC tumors [50], specifically in African American TNBC patients in comparison with non-Hispanic White TNBC patients [29]. In our study, we showed that the EV expression levels of miR-150-5p were different according to the BC subtypes investigated, being downregulated in TNBC compared to LA patients.

Higher expression levels of mir-320a in BC patients were previously correlated with improved overall survival [45]. Functional studies with cell lines described this miRNA as presenting anti-oncogenic activity, inhibiting cell proliferation, invasion [54,55], and metastasis [55,56].

Altogether, the patterns of miRNA expression in the observed studies, conducted in tumor tissues, do not necessarily correspond to what we observed in this study. This could be explained by the selective sorting of miRNAs into EVs [42,43]. Nevertheless, the associations that we found for all the EV-miRNAs investigated in the BC subtypes as well as clinicopathological parameters, indicate the potential prognostic value of these EV-miRNAs for BC patients. The diagnostic potential of three out of the four EV-miRNAs analyzed, presented significant accuracy for BC diagnosis, both individually and combined. The higher discriminatory value (AUC of 0.8387, sensitivity of 93.33% and specificity of 68.75%) obtained in the ROC analysis comparing CT versus CA, was from the panel constituted by the overexpression of miR-142-5p, miR-320a, and miR-4433b-5p. These values were higher than the previous study from Hannafon, et al. [7], which showed that an AUC value of 0.73 based on the combination of EV-miRNAs miR-21 and miR-1246, could be used to discriminate tumor from normal breast cells. When compared to other miRNA panels for BC diagnosis based on circulating miRNAs, our EV-miRNA panel outperforms them all [13,57,58].

Subtype classification is a key determinant for patient outcome and therapeutic choices. In this study, the analysis of EV-miRNA levels within two major BC groups, LA and TNBC, indicated that high expression levels of miR-142-5p were able to discriminate the LA patients from the TNBC patients with high accuracy (AUC = 0.9208, presenting 87.10% sensitivity and 81.25% specificity). This accuracy was further increased by the combination with miR-320a (AUC of 0.9410) which showed 100% sensitivity and 93.80% specificity, and thus presents a high potential as an EV-miRNA marker for the LA subtype.

## 5. Conclusions

Altogether, our results indicate the relevance of the four EV-miRNAs analyzed as diagnostic markers in BC. Additionally, they can be used to distinguish LA and TNBC subtypes. Importantly, the miRNA profiling of EVs isolated from liquid biopsies of BC using RNA-seq as a screening method, followed by RT-qPCR analysis of specific miRNAs can be used as a robust approach to identify miRNAs involved in cancer pathways. Results from these assays can be used, after further validation in a larger cohort, to augment the current BC diagnostic and prognostic methods available.

## Figures and Tables

**Table 1:** Clinicopathological parameters of breast cancer (BC) patients studied.

Parameters	BC Patients
Sample size (n)	31
Age (mean ± SD)	58.8 ± 15.5
Range in years	30–90
**Tumor Size (mm)**	
≤ 20	19
> 20	9
**Tumor Grade**	
I	4
II	11
III	13
**Lymph Node Metastasis**	
Positive	4
Negative	24
**Estrogen Receptor**	
Positive	16
Negative	15
**Progesterone Receptor**	
Positive	16
Negative	15
**HER2 Overexpression**	
Positive	0
Negative	31
**Proliferation Index (Ki-67)**	
≤ 14%	16
> 14%	13

HER2: human epidermal growth factor receptor 2.

**Table 2:** Top seven EV-miRNAs differentially expressed in RNA-seq analysis (screening phase) organized by adjusted *p*.

	EV-miRNAs	log2 FC	padj
**CT versus CA**	**hsa-miR-320a**	**−2.03**	**6.94 × 10^–5^**
	hsa-miR-126-5p	2.7	1.23 × 10^−4^
	hsa-miR-423-5p	–1.73	1.28 × 10^−3^
	hsa-miR-378a-3p	−2.29	1.64 × 10^−3^
	hsa-miR-185-5p	–1.81	1.64 × 10^−3^
	**hsa-miR-150-5p**	**2.47**	**1.64 × 10^−3^**
	hsa-miR-4454	2.93	2.14 × 10^−3^
**CT versus LA**	**hsa-miR-320a**	**−2.23**	**1.33 × 10^−6^**
	hsa-miR-423-5p	−1.91	2.06 × 10^−4^
	hsa-miR-744-5p	−1.75	4.67 × 10^−3^
	hsa-miR-103a-3p	−1.52	5.55 × 10^−3^
	hsa-miR-183-5p	1.4	6.55 × 10^−3^
	hsa-miR-126-5p	2.31	6.55 × 10^−3^
	hsa-let-7f-5p	1.23	1.20 × 10^−2^
**CT versus TNBC**	hsa-miR-185-5p	−2.2	5.26 × 10^−6^
	hsa-miR-195-5p	−2.74	5.94 × 10^−5^
	**hsa-miR-150-5p**	**3.4**	**5.94 × 10^−5^**
	hsa-miR-126-5p	3.35	6.82 × 10^−5^
	**hsa-miR-320a**	**−1.79**	**6.98 × 10^−5^**
	hsa-miR-26a-5p	2.18	6.98 × 10^−5^
	hsa-miR-4454	3.61	2.07 × 10^−3^
**LA versus TNBC**	**hsa-miR-4433b-5p**	**2.233**	**2.52 × 10^−5^**
	hsa-miR-26a-5p	1.768	8.62 × 10^−3^
	**hsa-miR-142-5p**	**1.853**	**1.83 × 10^−2^**
	hsa-let-7f-5p	−1.114	3.77 × 10^−2^
	hsa-miR-484	1.275	3.77 × 10^−2^
	hsa-miR-486-5p	1.252	4.17 × 10^−2^
	hsa-miR-15b-5p	2.39	4.77 × 10^−2^

The table shows differentially expressed miRNAs between groups. The EV-miRNAs selected for RT-qPCR analysis are underlined and highlighted in bold. CT (Control), LA (Luminal A), TNBC (Triple-Negative), CA (Cancer: LA + TNBC), FC (fold change), padj (adjusted *p*).

**Table 3:** Top ten Kyoto Encyclopedia of Genes and Genomes (KEGG) signaling pathway analyses of the four chosen EV-miRNAs. Pathways are listed according to the target-genes (#genes) of the four miRNAs (#miRNAs) analyzed.

KEGG Pathway	*p*-value	#genes	#miRNAs
Pathways in cancer	9.95 × 10^−5^	62	3
Viral carcinogenesis	3.10 × 10^−5^	42	3
Human T-cell lymphotropic virus type 1 (HTLV-I) infection	0.044937	40	3
MicroRNAs in cancer	0.038582	37	3
Mitogen-activated protein kinase (MAPK) signaling pathway	0.03917	37	3
Transcriptional misregulation in cancer	0.001051	33	3
Protein processing in endoplasmic reticulum	0.028384	31	3
Thyroid hormone signaling pathway	0.001618	29	3
Proteoglycans in cancer	0.004657	29	3
RNA transport	0.029074	28	3

**Table 4:** Diagnostic potential of the four EV-miRNAs, individually and combined, based on the ROC curve analysis.

EV-miRNA	Comparison	AUC	*p*-Value	Cutoff	Sensitivity	Specificity
miR-142-5p, miR-320a and miR-4433b-5p panel	CT versus CA	0.8387	<0.0001	0.4504	93.55%	68.75
miR-320a	CT versus CA	0.8063	<0.001	0.0060	93.33%	68.75%
miR-142-5p	CT versus CA	0.7964	<0.001	0.6435	87.10%	81.25%
miR-4433b-5p	CT versus CA	0.7964	<0.002	0.7578	87.10%	75%
miR-142-5p and miR-320a panel	CT versus LA	0.9410	<0.0001	0.0871	100%	93.80%
miR-142-5p	CT versus LA	0.9180	<0.0001	0.7926	100%	81.25%
miR-320a	CT versus LA	0.8828	<0.001	0.0065	93.75%	68.75%
miR-4433b-5p	CT versus LA	0.8672	<0.001	0.7743	93.75%	75%
miR-142-5p	TNBC versus LA	0.9208	<0.0001	0.6435	87.10%	81.25%
miR-150-5p	TNBC versus LA	0.8667	<0.001	39.3800	80%	75%

Only AUC > 0.79 values were represented.

## Supplementary Materials

The following are available online at https://www.mdpi.com/2218-273X/10/1/150/s1, **Table S1**: Complete list of DE EV-miRNAs found on RNA-seq analysis, separated by groups and organized according to *p*adj values. CT (control), LA (luminal A), TNBC (triple-negative), CA (cancer: LA + TNBC), **Table S2**: KEGG pathway analysis of DE EV-miRNAs separated by groups and classified according to the number of miRNAs.
