# Supplementary material for: Identification of miRNAs Enriched in Extracellular Vesicles Derived from Serum Samples of Breast Cancer Patients
Source: Biomolecules. 2020 Jan 16;10(1):150. doi: 10.3390/biom10010150 (PMC7022833; doi:10.3390/biom10010150)
Supplement: Supplementary file 1 [file biomolecules-10-00150-s001.pdf]

Table 1. Complete list of DE EV-miRNAs found on RNA-seq analysis, separated by groups and organized according to padj values. CT (control), LA (luminal A), TNBC (triple-negative), CA (cancer: LA + TNBC).

CT vs CA			CT vs LA			CT vs TNBC			LA vs TNBC		
**EV-miRNAs**	**log2 FC**	**padj**	**EV-miRNAs**	**log2FC**	**padj**	**EV-miRNAs**	**log2FC**	**padj**	**EV-miRNAs**	**log2FC**	**padj**
hsa-miR-320a	-2.03	6.94E-05	hsa-miR-320a	-2.23	1.33E-06	hsa-miR-185-5p	-2.20	5.26E-06	hsa-miR-4433b-5p	2.233	2.52E-05
hsa-miR-126-5p	2.70	1.23E-04	hsa-miR-423-5p	-1.91	2.06E-04	hsa-miR-195-5p	-2.74	5.94E-05	hsa-miR-26a-5p	1.768	8.62E-03
hsa-miR-423-5p	-1.73	1.28E-03	hsa-miR-744-5p	-1.75	4.67E-03	hsa-miR-150-5p	3.40	5.94E-05	hsa-miR-142-5p	1.853	1.83E-02
hsa-miR-378a-3p	-2.29	1.64E-03	hsa-miR-103a-3p	-1.52	5.55E-03	hsa-miR-126-5p	3.35	6.82E-05	hsa-let-7f-5p	-1.114	3.77E-02
hsa-miR-185-5p	-1.81	1.64E-03	hsa-miR-183-5p	1.40	6.55E-03	hsa-miR-320a	-1.79	6.98E-05	hsa-miR-484	1.275	3.77E-02
hsa-miR-150-5p	2.47	1.64E-03	hsa-miR-126-5p	2.31	6.55E-03	hsa-miR-26a-5p	2.18	6.98E-05	hsa-miR-486-5p	1.252	4.17E-02
hsa-miR-4454	2.93	2.14E-03	hsa-let-7f-5p	1.23	1.20E-02	hsa-miR-4454	3.61	2.07E-03	hsa-miR-15b-5p	2.39	4.77E-02
hsa-miR-103a-3p	-1.47	2.86E-03	hsa-miR-34a-5p	1.54	1.20E-02	hsa-miR-423-5p	-1.51	2.82E-03			
hsa-miR-320b	-1.75	6.80E-03	hsa-let-7b-5p	1.38	1.24E-02	hsa-miR-142-5p	1.97	2.82E-03			
hsa-miR-744-5p	-1.48	9.81E-03	hsa-miR-96-5p	2.37	1.24E-02	hsa-miR-16-5p	1.72	3.03E-03			
hsa-miR-5100	2.64	9.81E-03	hsa-miR-193a-5p	-2.47	2.08E-02	hsa-miR-103a-3p	-1.42	3.59E-03			
hsa-miR-96-5p	1.96	9.83E-03	hsa-miR-185-5p	-1.29	2.19E-02	hsa-miR-27a-3p	2.01	5.60E-03			
hsa-miR-193a-5p	-2.12	1.37E-02	hsa-miR-195-5p	1.18	2.82E-02	hsa-miR-378c	-3.75	5.88E-03			
hsa-miR-107	-1.35	1.37E-02	hsa-miR-150-5p	1.94	2.82E-02	hsa-miR-320b	-1.85	7.56E-03			
hsa-miR-183-5p	1.23	1.37E-02	hsa-miR-486-5p	-1.19	4.49E-02	hsa-miR-26b-5p	2.61	9.34E-03			
hsa-miR-27b-3p	2.18	1.37E-02	hsa-miR-4454	2.50	4.49E-02	hsa-miR-5100	3.10	1.09E-02			
hsa-miR-134-5p	-3.02	1.47E-02	hsa-miR-378a-3p	-1.64	4.50E-02	hsa-miR-107	-1.42	1.22E-02			
hsa-miR-320c	-1.68	1.50E-02	hsa-miR-320b	-1.62	4.50E-02	hsa-miR-126-3p	1.12	2.08E-02			
hsa-miR-320d	-1.72	1.56E-02	hsa-miR-107	-1.28	4.62E-02	hsa-miR-183-5p	1.07	2.51E-02			
hsa-miR-1307-3p	-1.47	2.54E-02				hsa-miR-4433b-5p	1.23	2.51E-02			
hsa-miR-378c	-2.99	2.74E-02				hsa-miR-320d	-1.85	2.79E-02			
hsa-miR-22-3p	-1.24	3.26E-02				hsa-miR-320c	-1.82	2.79E-02			
						hsa-miR-30b-5p	1.58	2.87E-02			
						hsa-miR-17-3p	-2.24	3.38E-02			
						hsa-miR-27b-3p	2.33	3.38E-02			
						hsa-miR-22-3p	-1.27	4.56E-02			
						hsa-miR-744-5p	-1.13	4.56E-02			

hsa-miR-335-5p	1.46	4.56E-02
hsa-miR-148a-3p	2.15	4.57E-02
hsa-miR-23b-3p	1.29	4.57E-02

Table 2. KEGG pathway analysis of DE EV-miRNAs separated by groups and classified according to the number of miRNAs.

	KEGG pathway	p-value	#genes	#miRNAs
CT vs CA	Proteoglycans in cancer	2.71E-15	127	21
	Colorectal cancer	1.69E-06	47	21
	Estrogen signaling pathway	6.67E-07	67	21
	Hippo signaling pathway	1.98E-07	91	21
	Protein processing in endoplasmic reticulum	6.53E-10	116	21
	Adherens junction	3.54E-10	58	20
	Viral carcinogenesis	1.11E-20	144	20
	Cell cycle	3.56E-08	86	19
	Renal cell carcinoma	3.81E-10	54	18
	Fatty acid metabolism	8.38E-08	26	17
CT vs LA	MicroRNAs in cancer	8.93E-67	130	19
	Proteoglycans in cancer	6.05E-17	147	19
	Viral carcinogenesis	3.98E-19	159	19
	Colorectal cancer	4.15E-09	55	19
	Adherens junction	1.19E-09	62	18
	Cell cycle	1.11E-14	104	18
	Non-small cell lung cancer	1.65E-08	48	18
	Prostate cancer	3.18E-09	76	18
	Renal cell carcinoma	2.23E-08	57	17
	Fatty acid metabolism	2.09E-11	32	16
CT vs TNBC	MicroRNAs in cancer	1.52E-47	145	29
	Pathways in cancer	4.56E-06	306	29
	Protein processing in endoplasmic reticulum	2.05E-07	142	29
	Proteoglycans in cancer	5.65E-11	169	29
	Chronic myeloid leukemia	5.97E-06	69	27
	Glioma	6.58E-10	60	27
	Renal cell carcinoma	2.05E-07	62	27
	Fatty acid metabolism	4.54E-07	36	25
	Fatty acid elongation	7.06E-07	20	24
	Glycosaminoglycan biosynthesis - keratan sulfate	1.76E-06	15	20
LA vs TNBC	Protein processing in endoplasmic reticulum	3.72E-11	97	6
	Adherens junction	2.96E-10	46	6
	Cell cycle	2.96E-10	74	6
	Lysine degradation	9.73E-09	28	6
	Proteoglycans in cancer	3.03E-07	97	6
	TGF-beta signaling pathway	8.83E-07	46	6
	Hippo signaling pathway	1.31E-06	66	6
	Oocyte meiosis	1.31E-06	58	6
	Renal cell carcinoma	1.31E-06	40	6
	Fatty acid biosynthesis	1.56E-06	6	5
